# Pituitary Apoplexy With Marked Hyperprolactinemia in a 19-Year-Old Woman: A Case Report of Diagnostic Challenges and Surgical Management

**DOI:** 10.7759/cureus.104700

**Published:** 2026-03-05

**Authors:** Zaid Al Hassani, Alaa H Al Masri, Mustafa Al Hassani, Ali Al Hassani, Rafi Khan

**Affiliations:** 1 General Medicine, Sheikh Tahnoon Medical City, Al Ain, ARE; 2 Internal Medicine, Sheikh Tahnoon Medical City, Al Ain, ARE

**Keywords:** adolescent, case report, hyperprolactinemia, pituitary adenoma, pituitary apoplexy, transsphenoidal surgery

## Abstract

Pituitary apoplexy is uncommon in adolescents and young adults and may present with subacute or evolving features rather than a fulminant endocrine emergency. Marked hyperprolactinemia in the setting of a hemorrhagic sellar mass can create diagnostic uncertainty between stalk effect and a prolactin-secreting tumor, with important implications for initial management.

A 19-year-old woman presented with a one-month history of progressive headaches and photophobia. Magnetic resonance imaging demonstrated a 15-mm hemorrhagic cystic pituitary lesion with suprasellar extension and mild displacement of the optic chiasm. Formal ophthalmologic assessment identified bilateral superior visual field defects. Serum prolactin was markedly elevated at 4624.73 mIU/L, while other pituitary hormones were within reference ranges. She was initially managed conservatively with stress-dose hydrocortisone and low-dose cabergoline under close clinical and visual monitoring. On day 5, she developed clinical deterioration with worsening headache, orthostatic symptoms, and blood pressure instability, prompting urgent endoscopic transsphenoidal resection with multilayer skull-base reconstruction. Postoperatively, she developed cerebrospinal fluid rhinorrhea that resolved with lumbar drainage, as well as transient diabetes insipidus treated with desmopressin. Prolactin fell rapidly to 153.47 mIU/L within 24 hours of surgery and normalized by six weeks. Histopathology confirmed a pituitary neuroendocrine tumor (PitNET). Headaches resolved completely within two weeks, and she returned to university studies within four weeks.

Pituitary apoplexy should be considered in young patients presenting with headache and visual disturbance, even with a subacute course. In hemorrhagic sellar lesions, markedly elevated prolactin may reflect either a prolactinoma or stalk effect; a rapid postoperative prolactin decline supports stalk compression as the dominant mechanism. Close observation during conservative management is essential, and clinical deterioration should prompt timely surgical decompression to optimize neurological and endocrine outcomes.

## Introduction

Pituitary apoplexy is a rare endocrine emergency characterized by acute hemorrhage or infarction within the pituitary gland, typically complicating a pre-existing adenoma, and producing a clinical spectrum ranging from asymptomatic to life-threatening manifestations [1-3]. Classic presentations include sudden severe headache, visual field defects, ophthalmoplegia, and altered consciousness [4]. Rapid recognition and management are critical due to the risk of acute adrenocorticotropic hormone (ACTH) deficiency precipitating adrenal crisis with hemodynamic instability, as well as neuro-ophthalmologic compromise from optic chiasm or cavernous sinus compression [3,4].

The incidence of pituitary apoplexy in adults with known pituitary adenomas ranges from 2% to 10%, predominantly affecting those aged 40-60 years with macroadenomas [5]. However, pituitary apoplexy is exceedingly rare in adolescents and young adults, occurring in only 1.8% to 7.5% of surgically treated pediatric pituitary neuroendocrine tumors (PitNETs), with a mean presentation age of 16.6 years [1]. This rarity reflects the low overall prevalence of pituitary adenomas in this age group, comprising fewer than 10% of all cases [3]. Notably, when apoplexy does occur in younger patients, it is more frequently associated with functioning adenomas, particularly prolactinomas (73.5% of pediatric cases), in contrast to adults, where non-functioning tumors predominate [1].

In adolescent females, marked hyperprolactinemia in a hemorrhagic sellar lesion may create diagnostic uncertainty between stalk effect and prolactinoma. Differentiation relies on the degree of prolactin elevation relative to tumor size, imaging findings, postoperative biochemical response, and histopathology when needed. We report a 19-year-old woman with a hemorrhagic pituitary lesion consistent with pituitary apoplexy who presented with subacute headaches and marked hyperprolactinemia and required endoscopic transsphenoidal resection. This case highlights the diagnostic challenge of distinguishing stalk effect from prolactinoma and the importance of close monitoring during initial conservative management.

## Case presentation

A 19-year-old woman presented to the outpatient clinic, accompanied by her mother, with a one-month history of intermittent occipital and frontal headaches interfering with sleep. The headaches were ongoing at presentation and were accompanied by photophobia; however, she denied visual field deficits, galactorrhea, or symptoms of thyroid dysfunction. Menstrual history revealed regular cycles until the preceding month, when intermittent bleeding occurred. Menarche occurred at age 12 years, and developmental milestones were normal. She was a university engineering student and a non-smoker, was not taking regular medications, and had no known drug allergies. Her past surgical history included a left tibia osteotomy. Family history was negative for pituitary or endocrine disorders.

Physical examination revealed an alert, oriented patient in no acute distress. Vital signs were: temperature 36.9 °C, heart rate 76 beats/minute, respiratory rate 18 breaths/minute, blood pressure 113/77 mmHg, oxygen saturation 99% on room air, height 167 cm, weight 81 kg (body mass index 29.0 kg/m²). Ophthalmologic assessment demonstrated pupils that were equal, round, and reactive to light, with no visual field defects on confrontation testing. Neurological examination showed normal sensory and motor function, with no clinical features of cortisol or growth hormone excess. Cardiovascular, respiratory, abdominal, and musculoskeletal examinations were unremarkable.

Brain magnetic resonance imaging (MRI), performed one day before admission, demonstrated a 15-mm hemorrhagic cystic pituitary adenoma, prompting hospital admission for further evaluation.

The patient was admitted on day 0, hemodynamically stable, with a hemorrhagic sellar lesion concerning for evolving pituitary apoplexy. Initial assessment showed a random serum cortisol of 202 nmol/L, which did not indicate overt adrenal insufficiency at presentation. However, because pituitary apoplexy can evolve dynamically with developing ACTH deficiency, empirical stress-dose hydrocortisone was initiated as a precaution while close clinical and biochemical monitoring continued. Initial complete blood count and renal function were within reference ranges (Table 1). A comprehensive pituitary hormonal profile was planned on admission and completed shortly thereafter as part of the formal endocrine evaluation (Table 2). By day 2, pituitary testing demonstrated markedly elevated prolactin at 4624.73 mIU/L (reference range 102-496 mIU/L), while the remaining anterior pituitary hormones were within reference ranges (Table 2).

**Table 1:** Initial admission laboratory results (day 0) before comprehensive pituitary hormonal profiling.

Test	Result	Reference range
Hemoglobin	134 g/L	120-160 g/L
White cell count	7.2 × 10⁹/L	4.0-11.0 × 10⁹/L
Platelets	245 × 10⁹/L	150-400 × 10⁹/L
Serum creatinine	62 μmol/L	45-84 μmol/L
Estimated glomerular filtration rate	>90 mL/min/1.73 m²	>90 mL/min/1.73 m²
Random serum cortisol	202 nmol/L	138-690 nmol/L

**Table 2:** Comprehensive pituitary hormone profile obtained on hospital day 2. FSH, follicle-stimulating hormone; LH, luteinizing hormone; TSH, thyroid-stimulating hormone; GH, growth hormone; IGF‑1, insulin-like growth factor 1; T3, triiodothyronine; T4, thyroxine

Hormone	Result	Reference range (Approximate)
FSH (mIU/mL)	2.99	1.5-12.4 (follicular phase)
LH (mIU/mL)	2.15	1.9-12.5 (follicular phase)
Prolactin (mIU/L)	4624.73	102-496
Estradiol (pmol/L)	125	70-500 (follicular phase)
TSH (mIU/mL)	0.620	0.270-4.200
Free T3 (pmol/L)	3.37	3.10-6.80
Free T4 (pmol/L)	13.89	12.00-22.00
GH (mIU/mL)	3.12	<5.0 (random)
IGF-1 (nmol/L)	54.10	11.6-306 (age-adjusted)

**Figure 1:**
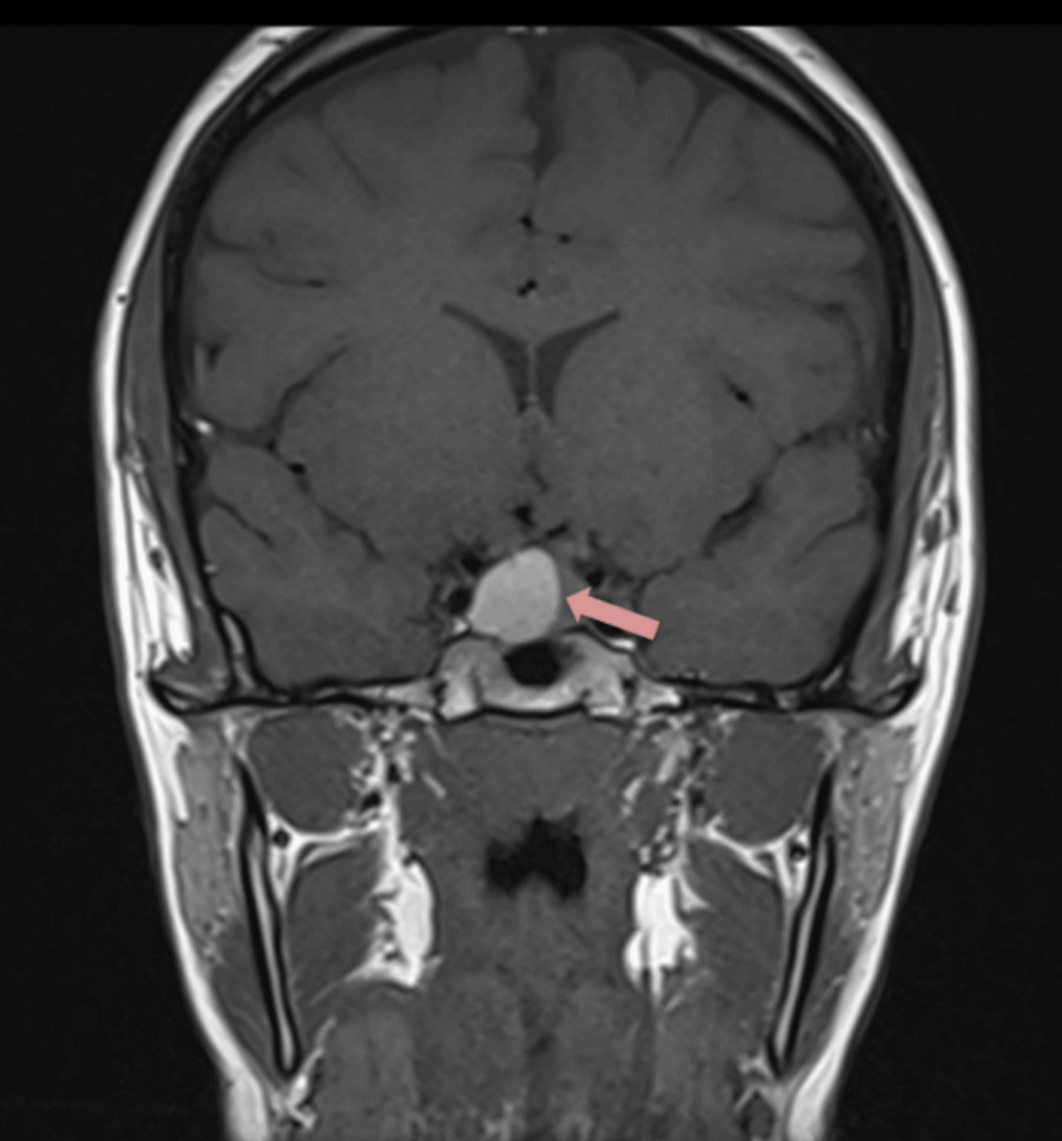
Contrast-enhanced pituitary magnetic resonance imaging (MRI) demonstrating pituitary apoplexy (arrow). Dedicated pituitary MRI with contrast shows a 12 × 13 × 15 mm cystic sellar lesion with suprasellar extension and mild superior displacement of the optic chiasm. The lesion demonstrates a fluid-fluid level and hemorrhagic signal characteristics without significant post-contrast enhancement. The pituitary stalk is deviated leftward, the cavernous sinuses are unremarkable, and internal carotid artery flow voids are preserved.

Urgent pituitary MRI with contrast confirmed a 12 × 13 × 15 mm cystic lesion with suprasellar extension and mild displacement of the optic chiasm. The lesion demonstrated a fluid-fluid level with hyperintense signal anteriorly on T1- and T2-weighted sequences, intermediate-to-low signal posteriorly, leftward deviation of the pituitary stalk, and no significant post-contrast enhancement, findings consistent with pituitary apoplexy (Figure 1).

The differential diagnosis at presentation included Rathke cleft cyst apoplexy, hemorrhagic pituitary macroadenoma, and craniopharyngioma. Rathke cleft cyst apoplexy was considered, given the cystic nature and fluid-fluid level on imaging. Hemorrhagic pituitary macroadenoma remained the leading diagnosis given the suprasellar extension, optic chiasm displacement, and extreme hyperprolactinemia. Craniopharyngioma was considered but deemed less likely given the patient’s age and imaging characteristics, lacking calcification. The combination of hemorrhagic signal characteristics, marked hyperprolactinemia, and clinical presentation supported pituitary apoplexy as the primary diagnosis.

On day 1, a formal ophthalmologic assessment revealed bilateral superior visual field defects on automated perimetry. Neurosurgery recommended conservative observation with daily visual field monitoring and comprehensive pituitary profiling. Endocrinology initiated intravenous hydrocortisone 100 mg stat followed by 50 mg every six hours, oral cabergoline 0.25 mg weekly, and strict fluid balance monitoring for diabetes insipidus.

Additional investigations, including N-terminal pro-B-type natriuretic peptide, C-reactive protein, procalcitonin, lipid profile, and liver function tests, were within reference limits (Table 3).

**Table 3:** Additional laboratory results on day 2. NT-proBNP, N-terminal pro-B-type natriuretic peptide; HDL, high-density lipoprotein; LDL, low-density lipoprotein

Test	Result	Reference range
NT-proBNP (ng/L)	21.2	<125
C-reactive protein (mg/L)	Normal	<10
Procalcitonin (ng/mL)	Normal	<0.25
Total cholesterol (mmol/L)	4.57	<5.2
HDL cholesterol (mmol/L)	1.87	1.0
LDL cholesterol (mmol/L)	2.69	<3.0
Triglycerides (mmol/L)	0.23	<1.7
Cholesterol/HDL ratio	2.44	<5.0

The markedly elevated prolactin level (>4,000 mIU/L) raised consideration of prolactinoma, although such elevations can also occur secondary to pituitary stalk compression from non-functioning adenomas or other sellar masses, representing a diagnostic challenge requiring histopathological confirmation.

On days 3 and 4, hydrocortisone was tapered to 50 mg intravenously three times daily on day 3, then to 20 mg orally three times daily on day 4. Neurosurgery maintained conservative management with analgesic support for headache control.

**Figure 2:**
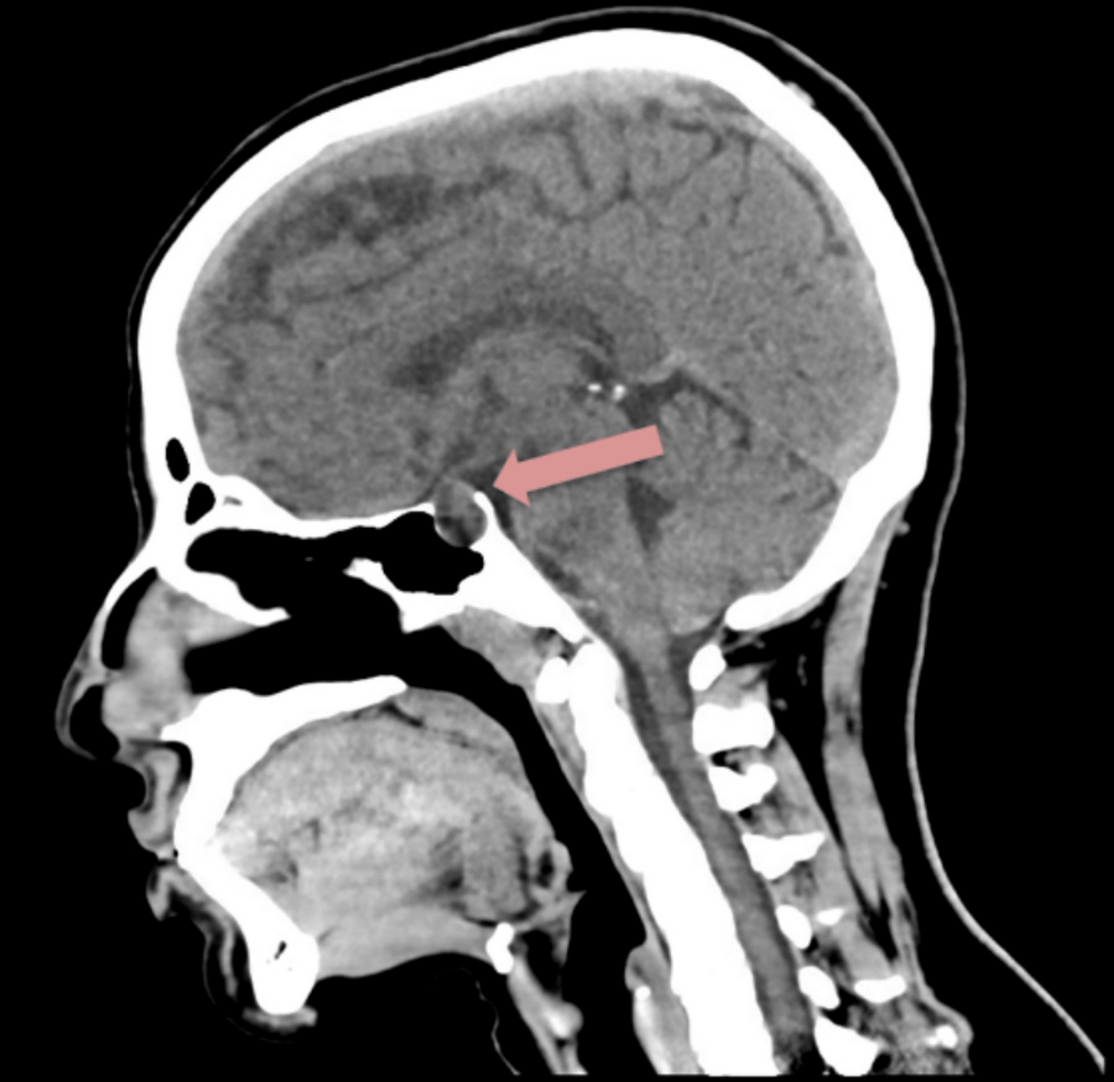
Preoperative navigation computed tomography showing a hemorrhagic sellar lesion with suprasellar extension (arrow). The scan demonstrates a heterogeneous sellar mass with suprasellar extension and mild displacement of the optic chiasm, consistent with pituitary apoplexy.

On day 5, the patient reported worsening occipital and retroorbital headaches, exacerbated by eating and with early-morning predominance, as well as orthostatic dizziness. Blood pressure fluctuated between 106/62 and 116/80 mmHg. Intravenous fluid hydration was increased. Urgent neurosurgical consultation recommended proceeding with endoscopic transsphenoidal resection. Navigation computed tomography confirmed a hemorrhagic lesion with heterogeneous attenuation and mass effect on the optic chiasm (Figure 2).

On day 6 (surgical intervention), following comprehensive preoperative counseling regarding surgical risks, including hypopituitarism, diabetes insipidus, cerebrospinal fluid (CSF) leak, visual complications, and vascular injury, the patient provided informed consent. 

Endoscopic transsphenoidal resection was performed under general anesthesia with neuronavigation guidance. A right-sided endonasal approach with wide sphenoidotomy allowed access to the sella, where dural opening released a high-pressure liquefied hematoma. The hemorrhagic adenoma was resected with preservation of normal pituitary tissue and clear margins. Multilayer sellar reconstruction was undertaken using a dural substitute, vascularized nasoseptal flap, hemostatic agents, autologous fat, and balloon support. No intraoperative complications occurred.

Postoperative management included intensive care unit admission with hourly neurological observations, serial sodium monitoring every 6 hours, strict fluid balance charting, and withholding corticosteroids pending morning cortisol assessment. Intravenous cefepime and vancomycin were administered in accordance with the local perioperative neurosurgical protocol for infection prophylaxis after endoscopic transsphenoidal surgery and skull-base reconstruction, rather than as a universal standard regimen. Postoperative computed tomography demonstrated expected changes with no residual hematoma or other acute intracranial complication.

On day 7 (postoperative day 1), the patient was alert with a Glasgow Coma Scale score of 15/15. Clear CSF rhinorrhea developed from the right nostril, representing an adverse postoperative event. Repeat pituitary hormone profiling showed prolactin decreased to 153.47 mIU/L, with other pituitary hormones and cortisol (on replacement) within acceptable ranges (Table 4). Serum sodium remained stable at 138 mmol/L (136-145 mmol/L).

**Table 4:** Pituitary hormone profile on postoperative day 1 (day 7). FSH, follicle-stimulating hormone; LH, luteinizing hormone; TSH, thyroid-stimulating hormone; GH, growth hormone; IGF‑1, insulin-like growth factor 1; T3, triiodothyronine; T4, thyroxine

Hormone	Result	Reference range (approximate)
FSH (mIU/mL)	5.35	1.5-12.4 (follicular phase)
LH (mIU/mL)	4.95	1.9-12.5 (follicular phase)
Prolactin (mIU/L)	153.47	102-496
Estradiol (pmol/L)	<88.0	70-500 (follicular phase)
TSH (mIU/mL)	2.030	0.270-4.200
Free T3 (pmol/L)	2.91	3.10-6.80
Free T4 (pmol/L)	14.52	12.00-22.00
Cortisol (nmol/L)	717.7 (on hydrocortisone)	138-690 (morning)
GH (mIU/mL)	14.30	<5.0 (random)
IGF-1 (nmol/L)	37.80	11.6-306 (age-adjusted)

An urgent lumbar drain was inserted by interventional radiology, draining 15 mL/hour. CSF analysis revealed glucose 3.8 mmol/L, protein 0.35 g/L, 2 white cells/μL, and 5 red cells/μL, with negative Gram stain and culture (Table 5).

**Table 5:** Cerebrospinal fluid analysis on postoperative day 1 (day 7).

Parameter	Result	Reference range
Glucose	3.8 mmol/L	2.5-4.5 mmol/L
Protein	0.35 g/L	0.15-0.45 g/L
White blood cells	2 cells/μL	0-5 cells/μL
Red blood cells	5 cells/μL	0-5 cells/μL
Gram stain	Negative	Negative
Culture	No growth	No growth

On day 8 (postoperative day 2), the Glasgow Coma Scale remained 15/15 with resolution of CSF leak. The patient was transferred to the general ward with a lumbar drain and a nasal catheter maintained. Hydrocortisone was reduced to 50 mg intravenously three times daily. Polyuria developed later (2,000 mL over six hours) with serum sodium 141 mmol/L, consistent with transient diabetes insipidus. Intravenous desmopressin 1 μg twice daily was commenced, with dose titration based on urine output and osmolality.

On days 11 through 14 (postoperative days 5-8), the patient was transferred to the ward on day 11 with transition to oral desmopressin 60 μg at bedtime, and the lumbar drain rate was decreased to 10 mL/hour, then 5 mL/hour on day 12. Desmopressin was discontinued with as-needed dosing parameters established (if urine output exceeded 250 mL/hour or sodium exceeded 150 mmol/L). Hydrocortisone was tapered to 25 mg orally three times daily, then 20 mg twice daily. The lumbar drain was disconnected on day 13 and removed on day 14 without complications. Desmopressin 60 μg at bedtime was resumed, and the patient was discharged in stable condition with outpatient follow-up arranged.

Examination of surgical specimens revealed a pituitary neuroendocrine tumor with a disrupted reticulin pattern, small-to-medium cells with round nuclei, no atypia, inconspicuous mitoses, and no necrosis. Immunohistochemistry showed diffuse synaptophysin and perinuclear CAM 5.2 positivity, negative chromogranin, cytokeratins, glial fibrillary acidic protein, thyroid transcription factor-1, and caudal-type homeobox 2, with retained INI1 and a Ki-67 proliferation index of approximately 2%, confirming pituitary adenoma.

At four weeks post-discharge, desmopressin was increased to 60 μg twice daily for persistent polyuria, and hydrocortisone was discontinued. At six weeks, the multidisciplinary tumor board recommended a 3-month postoperative MRI, repeat hormone assessment, histopathological immunostaining for pituitary hormones (prolactin, adrenocorticotropic hormone, growth hormone, follicle-stimulating hormone, luteinizing hormone, thyroid-stimulating hormone), transcription factors (steroidogenic factor-1, Pit-1, T-pit), and genetic testing, including AIP gene mutation analysis.

The patient reported complete resolution of headaches by two weeks post-discharge and successfully returned to university studies by four weeks postoperatively. She demonstrated excellent medication adherence, assessed by self-report and pharmacy records. Cabergoline was well tolerated, with no nausea or orthostatic symptoms at the low dose of 0.25 mg weekly. Desmopressin titration was guided by patient-reported urine output and clinic sodium measurements, with good tolerability throughout.

At final follow-up six weeks after discharge, paired urine and plasma osmolality were within reference ranges, pituitary hormones demonstrated satisfactory recovery with prolactin normalized, and diabetes insipidus had resolved with desmopressin discontinued. The patient was referred to clinical genetics for counseling and AIP mutation testing. Histopathology immunostaining was requested to determine adenoma subtype, and a three-month postoperative MRI was scheduled with ongoing endocrinology surveillance.

A comprehensive timeline is presented in Table 6.

**Table 6:** Timeline of clinical course, investigations, and management PitNET, pituitary neuroendocrine tumor; CSF, cerebrospinal fluid

Time point	Events, assessments, and key results	Management and outcomes
~1 month before admission	Onset of intermittent occipital and frontal headaches affecting sleep, associated with photophobia	Symptomatic course continued until presentation
Day 1	Outpatient brain MRI showed a 15 mm hemorrhagic cystic pituitary lesion with suprasellar extension.	Admission arranged for evaluation and monitoring
Day 0-1	Admitted with suspected pituitary apoplexy. Random cortisol 202 nmol/L. Pituitary MRI with contrast: 12 × 13 × 15 mm cystic sellar lesion with suprasellar extension, mild optic chiasm displacement, fluid-fluid level, no enhancement, leftward stalk deviation. Bilateral superior visual field defects on formal testing	Conservative management: intravenous hydrocortisone, oral cabergoline, daily visual assessment, and monitoring for diabetes insipidus
Days 2-4	Pituitary profile: prolactin 4624.73 mIU/L, other anterior pituitary hormones within range. Additional labs normal	Hydrocortisone tapered to oral regimen. Conservative management continued with close clinical and visual monitoring.
Days 5-6	Worsening occipital and retroorbital headaches, orthostatic dizziness, and blood pressure variability. Navigation CT confirmed sellar lesion with suprasellar extension and mild optic chiasm displacement.	Urgent neurosurgical review. Endoscopic transsphenoidal resection with multilayer reconstruction performed on day 6
Day 7 (postoperative day 1)	Glasgow Coma Scale 15/15. Clear CSF rhinorrhea from the right nostril. Prolactin 153.47 mIU/L; serum sodium 138 mmol/L. CSF analysis was normal.	Lumbar drain inserted (15 mL/hour). Hydrocortisone continued intravenously
Day 8 (postoperative day 2)	CSF leak resolved. Polyuria (2,000 mL over six hours) with serum sodium 141 mmol/L, consistent with transient diabetes insipidus	Transferred to the ward. Lumbar drain and nasal catheter maintained. Intravenous desmopressin started and titrated; hydrocortisone dose reduced.
Days 11-14 (postoperative days 5-8)	Ongoing clinical stability. Improving fluid balance	Lumbar drain was weaned and removed. Transition to oral desmopressin with as‑needed parameters and tapered oral hydrocortisone. Discharged in stable condition
Histopathology (day 6 specimen)	Pituitary neuroendocrine tumor (adenoma) with a Ki-67 index of approximately 2% and an immunoprofile consistent with PitNET	The multidisciplinary tumor board recommended further hormone/transcription factor immunostaining and AIP mutation testing.
Weeks 4-6 post‑discharge	Complete resolution of headaches by two weeks. Return to university by four weeks. Prolactin was normalized. Diabetes insipidus resolved; desmopressin discontinued	Hydrocortisone was discontinued. Ongoing endocrinology follow‑up with planned three‑month postoperative MRI and clinical genetics evaluation
~1 month before admission	Onset of intermittent occipital and frontal headaches affecting sleep, associated with photophobia	Symptomatic course continued until presentation

## Discussion

Pathophysiologically, pituitary apoplexy results from rapid tumor expansion with compromised vascular supply, compression of the superior hypophyseal arterial branches, and inherent vascular fragility, leading to acute ischemia followed by hemorrhage [4,6]. MRI is the diagnostic modality of choice, characteristically revealing hemorrhagic components, fluid-fluid levels, and sellar mass effect, while formal visual field testing (e.g., Humphrey automated perimetry) is essential for quantifying neuro-ophthalmologic deficits [3,4].

This patient’s clinical presentation demonstrated both typical and atypical features of pituitary apoplexy. While apoplexy classically presents with sudden severe headache, this patient experienced subacute intermittent headaches over one month before clinical deterioration, representing an atypical temporal pattern [1]. Subacute presentations of hemorrhage or infarction into the pituitary have a similar natural history and outcomes to classic acute apoplexy, suggesting that these disparate presentations represent a spectrum of the same condition [5]. The worsening headache pattern, orthostatic symptoms, and blood pressure variability on day 5 prompted urgent surgical intervention, aligning with evidence that deteriorating clinical status warrants early decompression. Her bilateral superior visual field defects on formal perimetry and mild optic chiasm displacement corroborated the radiological diagnosis, consistent with approximately 75% of apoplexy cases that present with visual compromise [7].

The markedly elevated prolactin level of 4624.73 mIU/L presented a diagnostic dilemma central to this case. Traditional teaching suggests that prolactin levels exceeding 500 µg/L (approximately 10,870 mIU/L using the conversion factor 1 µg/L = 21.7 mIU/L) are highly specific for prolactinomas (98% specificity), whereas moderate elevations can result from pituitary stalk compression in non-functioning adenomas, termed the *stalk effect* [8]. This patient’s prolactin level of 4624.73 mIU/L (approximately 213 ng/mL) fell into an ambiguous range in which both prolactinoma and stalk compression from a non-functioning adenoma with apoplectic hemorrhage remained plausible. Although the prolactin-per-unit-tumor-volume ratio may improve diagnostic discrimination in selected cases, a reliable calculation was not possible here because the lesion was predominantly hemorrhagic and cystic, and accurate measurement of the solid tumor component was not available on the imaging reviewed. Accordingly, postoperative biochemical behavior and histopathologic assessment were more informative in distinguishing stalk effect from a prolactin-secreting tumor in this case. Recent volumetric analyses demonstrate that the prolactin-per-unit-tumor-volume ratio can distinguish prolactinomas from stalk effect with greater accuracy than absolute prolactin values alone [9]. In non-functioning pituitary macroadenomas, preoperative prolactin concentrations less than 2.35-fold above normal predict 95.2% normalization rates after decompression, whereas higher elevations suggest mixed pathology [10]. In this case, the rapid prolactin decline from 4624.73 mIU/L to 153.47 mIU/L within 24 hours postoperatively, followed by normalization by six weeks, supported stalk compression as the predominant mechanism rather than a true prolactin-secreting tumor, although final histopathological hormone immunostaining remains pending.

Management of pituitary apoplexy remains controversial, with ongoing debate between conservative therapy and surgical intervention [11]. Recent meta-analyses demonstrate that surgical intervention is more effective than conservative approaches in improving ocular palsy recovery rates, although no significant differences have been observed in endocrine function recovery between groups [11]. Current guidelines recommend conservative management for patients with isolated headache or mild, non-progressive visual impairment, while advocating early surgical intervention for those with moderate-to-severe visual deficits or significant mass effect on imaging [11]. This patient initially received conservative management with stress-dose hydrocortisone and cabergoline, consistent with her stable presentation and only mild visual field defects on day 0. However, clinical deterioration on day 5, with worsening headache pattern, orthostatic symptoms, and blood pressure instability, prompted urgent surgical decompression. This clinical trajectory aligns with evidence supporting tailored approaches that monitor for deterioration and intervene surgically when neurological status declines [11]. Endoscopic transsphenoidal resection with multilayer skull-base reconstruction was performed, preserving normal pituitary tissue while achieving complete adenoma resection with clear margins.

A postoperative CSF leak occurred on day 7. The introduction of vascularized nasoseptal flaps has reduced CSF leak rates after endoscopic endonasal approaches from as high as 24% in early series to as low as 3% in recent studies [12]. Despite intraoperative multilayer closure, including a nasoseptal flap, postoperative CSF rhinorrhea developed and was successfully managed with lumbar drainage at 15 mL/hour, achieving complete resolution within 24 hours, with drain removal on day 14 and no recurrence. Transient diabetes insipidus developed on postoperative day 2, manifesting with polyuria. Postoperative diabetes insipidus occurs in approximately 13% to 16% of patients undergoing endoscopic pituitary surgery, with most cases being transient and resolving spontaneously or with brief treatment [13,14]. This patient’s diabetes insipidus was successfully managed with desmopressin dose titration based on urine output and serum sodium monitoring, achieving complete resolution by six weeks post-discharge without the need for long-term therapy, consistent with the transient pattern in which vasopressin deficiency resolves within days to weeks postoperatively.

Clinical outcomes were excellent following surgical decompression. Visual recovery was rapid, with complete resolution of bilateral superior visual field defects, consistent with literature demonstrating visual improvement in 75% to 85% of pituitary apoplexy patients after surgery [4]. Prolactin declined dramatically from 4624.73 mIU/L preoperatively to 153.47 mIU/L within 24 hours post-surgery and normalized by six weeks postoperatively without dopamine agonist therapy. This rapid normalization aligns with evidence that 77.8% of patients with stalk effect-related hyperprolactinemia experience prolactin normalization after transsphenoidal decompression [15]. Headaches resolved completely within two weeks, and the patient returned to university studies with full functional independence. The favorable outcome in this young patient is consistent with emerging evidence suggesting that pediatric and adolescent pituitary apoplexy may have more favorable neurological and endocrine outcomes than adult cases [16].

Multidisciplinary involvement from neurosurgery, endocrinology, and ophthalmology enabled comprehensive hormonal assessment and formal visual field testing. Escalation to surgical management on day 5, when clinical deterioration occurred, supported favorable visual and endocrine outcomes. Despite postoperative CSF leak, multilayer skull-base reconstruction achieved complete healing without long-term sequelae.

Limitations include the inherent lack of generalizability of a single case report, pending hormone immunohistochemistry precluding definitive tumor subtyping, and genetic testing for familial adenoma syndromes not yet completed. In addition, the current follow-up duration of six weeks is relatively short for a young patient with a pituitary tumor and limits assessment of long-term recurrence risk, residual tumor behavior, and delayed pituitary dysfunction. Long-term surveillance is therefore planned, including the already scheduled three-month postoperative MRI, repeat endocrine testing, and continued multidisciplinary endocrinology and neurosurgical follow-up, with subsequent radiologic and hormonal monitoring guided by postoperative findings and clinical course.

## Conclusions

This case highlights that pituitary apoplexy can present subacutely in young patients and should be considered when a persistent headache is accompanied by visual field changes and a hemorrhagic sellar lesion. Marked hyperprolactinemia in this setting can reflect either stalk effect or prolactinoma; in our patient, the rapid postoperative fall in prolactin supported stalk compression as the predominant mechanism. Close monitoring is essential during initial conservative management, and clinical deterioration should prompt timely surgical decompression. With multidisciplinary care, surgery and postoperative complications were managed successfully, leading to rapid symptomatic and endocrine recovery.
